# Musashi1 Contribution to Glioblastoma Development via Regulation of a Network of DNA Replication, Cell Cycle and Division Genes

**DOI:** 10.3390/cancers13071494

**Published:** 2021-03-24

**Authors:** Mirella Baroni, Caihong Yi, Saket Choudhary, Xiufen Lei, Adam Kosti, Denise Grieshober, Mitzli Velasco, Mei Qiao, Suzanne S. Burns, Patricia R. Araujo, Talia DeLambre, Mi Young Son, Michelina Plateroti, Marco A. R. Ferreira, E. Paul Hasty, Luiz O. F. Penalva

**Affiliations:** 1 Children’s Cancer Research Institute, UT Health San Antonio, San Antonio, TX 78229, USA; mibaroni@alumni.usp.br (M.B.); ych14789850262@sina.com (C.Y.); lei@uthscsa.edu (X.L.); kosti@uthscsa.edu (A.K.); denisegrieshober@freenet.de (D.G.); mitzvelo@gmail.com (M.V.); ljqmlx@hotmail.com (M.Q.); biopaty@hotmail.com (P.R.A.); takaviol@gmail.com (T.D.); 2 Third Xiangya Hospital, Central South University, Changsha 410000, China; 3 Computational Biology and Bioinformatics, University of Southern California, Los Angeles, CA 90089, USA; skchoudh@usc.edu; 4 Department of Cell Systems and Anatomy, UT Health San Antonio, San Antonio, TX 78229, USA; 5 Department of Molecular Medicine, Sam and Ann Barshop Institute for Longevity and Aging Studies, UT Health San Antonio, San Antonio, TX 78229, USA; sonmy@uthscsa.edu (M.Y.S.); hastye@uthscsa.edu (E.P.H.); 6 Team: Development, Cancer and Stem Cells, Université de Strasbourg, Inserm, IRFAC/UMR-S1113, FMTS, 67200 Strasbourg, France; plateroti@unistra.fr; 7 Department of Statistics, Virginia Tech, Blacksburg, VA 24060, USA; marf@vt.edu

**Keywords:** glioblastoma, Musashi1, cell cycle, DNA replication, cell division, E2F2, E2F8

## Abstract

**Simple Summary:**

Glioblastoma (GBM) is one of the most aggressive tumor types with no effective treatment options. To create new routes for therapy, it is necessary to continue mapping new pathways contributing to gliomagenesis. In this regard, there is growing evidence that RNA binding proteins (RBPs) are major contributors to expression alterations affecting genes in signaling pathways critical to GBM growth and response to therapy. We have established Musashi1 (Msi1) as a main player in GBM and medulloblastoma and as a marker of clinical outcome and response to therapy. Our genomic and functional analyses established that Msi1 directly and indirectly regulates the expression of a network of genes, promoting cell cycle progression and DNA replication. Ultimately, Msi1 impact on this network has important consequences in tumor initiation, growth and response to therapy.

**Abstract:**

RNA-binding proteins (RBPs) function as master regulators of gene expression. Alterations in their levels are often observed in tumors with numerous oncogenic RBPs identified in recent years. Musashi1 (Msi1) is an RBP and stem cell gene that controls the balance between self-renewal and differentiation. High Msi1 levels have been observed in multiple tumors including glioblastoma and are often associated with poor patient outcomes and tumor growth. A comprehensive genomic analysis identified a network of cell cycle/division and DNA replication genes and established these processes as Msi1’s core regulatory functions in glioblastoma. Msi1 controls this gene network via two mechanisms: direct interaction and indirect regulation mediated by the transcription factors E2F2 and E2F8. Moreover, glioblastoma lines with Msi1 knockout (KO) displayed increased sensitivity to cell cycle and DNA replication inhibitors. Our results suggest that a drug combination strategy (Msi1 + cell cycle/DNA replication inhibitors) could be a viable route to treat glioblastoma.

## 1. Introduction

Glioblastoma (GBM) is one of the most aggressive tumor types with no effective treatment options. In The Cancer Genome Atlas (TCGA), hundreds of GBM tumors were analyzed for DNA copy number, DNA methylation, and gene expression [1]. The results of these analyses largely increased our knowledge and helped improve GBM classification. However, therapeutic strategies based on these new findings have not yet become a reality. To map new pathways contributing to GBM development and to potentially create new routes for therapy, we turned our attention to oncogenic RNA binding proteins (RBPs) and their network of targets. Over 1500 RBPs have been cataloged in the human genome [2]. Changes in their levels or functional activity can lead to multiple alterations in RNA processing and expression that can eventually contribute to the acquisition of cancer-relevant phenotypes [3]. RBPs are particularly relevant in the nervous system where splicing and translation regulate critical aspects of neurogenesis, neural function, and nervous system development [4]. Not surprisingly, RBPs have been shown to be important oncogenic factors in medulloblastoma and gliomas [5]. In recent years, several studies have established the feasibility of developing specific inhibitors against RBPs based on the uniqueness of their RNA binding domains. RBP inhibition supposedly could affect multiple oncogenic pathways at once, providing a robust alternative in cancer therapy [6].

We have identified the stem cell RBP Musashi1 (Msi1) as a key contributor to brain tumor (GBM and medulloblastoma) development and as a marker of clinical outcome and response to therapy [2,3,4,7,8,9,10]. Msi1 role as an onco-factor is supported by numerous studies in liver, pancreas, breast, lung, colon, and many other tumors [5,11]. Moreover, Msi1 has been linked to chemoresistance in different tumors [12,13,14,15,16,17]. In GBM, Msi1 overexpression promotes a protective effect in drug-induced cell death, thus facilitating the formation of chemo-resistant stress granules after treatment [16] or through down-regulating pro-apoptotic genes [18]. While in medulloblastoma, Msi1 knockdown increased sensitivity to cyclopamine, an SHH inhibitor [17]. Msi1 positive cells have been shown to be resistant to oxaliplatin and 5-fluorouracil in colon cancer [13]. Msi1 function is also critical to the metastasis process in breast cancer [19] and metastatic samples prevalently display high Msi1 expression [20]. Increased Msi1 expression might also be linked to tumor initiation since its expression is required for the survival of cancer stem cells, maintenance of the stem cell signature, and promotion of oncogenic activation and cell cycle progression [3,9,11,20,21,22].

Msi1 regulates gene expression mainly by binding to specific sequences in the 3′UTR of its target genes (transcripts), causing changes in mRNA stability and translation [5,12]. Functional genomic studies showed that Msi1 regulates a large number of transcripts associated with multiple pathways implicated in tumorigenesis [2,3,4,5,8,9,10,11,20,21,22,23]. To establish Msi1’s main contributions to glioblastoma development, we analyzed U251 and U343 Msi1 knockout (KO) lines and their controls by RNA-seq and conducted then an integrated analysis, using Msi1 expression profiles and other genomic datasets. DNA replication and cell cycle/division were identified as the processes affected by Msi1 the most and biological analyses corroborated Msi1 impact on these processes. Msi1 regulates this network of genes directly and indirectly, having the transcription factors E2F2 and E2F8 as main mediators. Ultimately, Msi1’s impact on this network has important consequences to tumor initiation, growth, and response to therapy. Importantly, glioblastoma cells with Msi1 KO showed increased sensitivity to cell cycle and DNA replication inhibitors. This result opens the possibility of using drug combinations (Msi1 and cell cycle/DNA replication inhibitors) to treat GBM patients.

## 2. Results

### 2.1. Genomic Analyses Define Regulation of DNA Replication and Cell Cycle/Division as Musashi1 Core Functions

**Figure 1:**
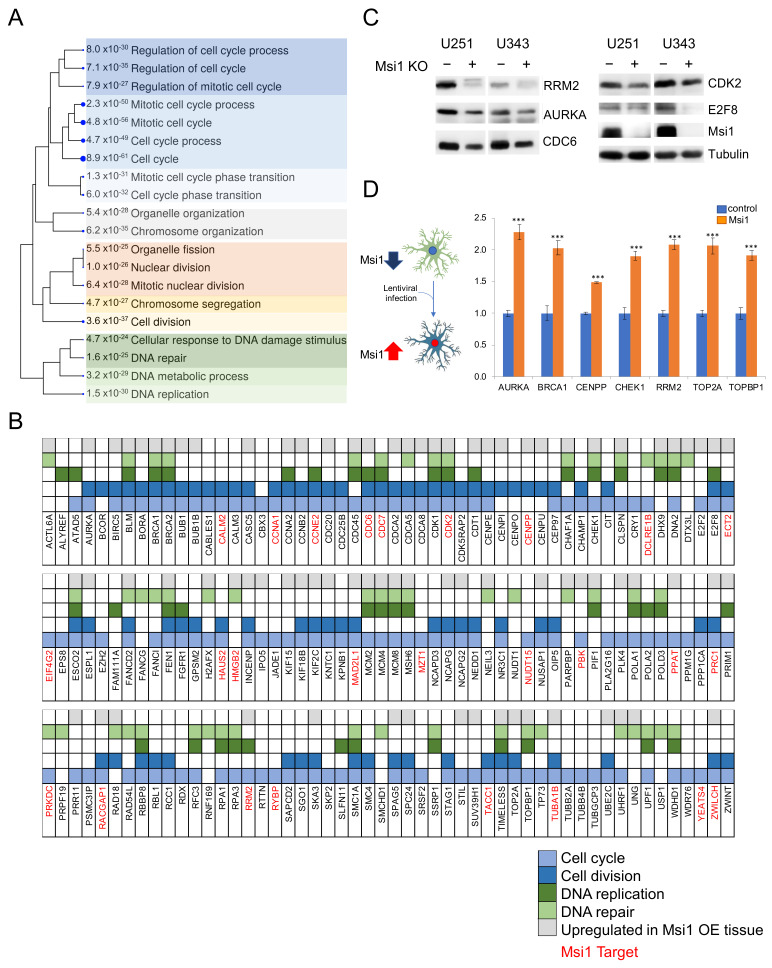
Musashi1 knockout (KO) affects a network of genes implicated in cell cycle/division and DNA replication/repair. (**A**) The diagram shows the Gene Ontology terms (biological processes) enriched among genes downregulated in both U251 and U343 Msi1 KO lines in comparison to respective controls, according to ShinyGo [27]. (**B**) Tables show downregulated genes in both U251 and U343 Msi1 KO lines and their functions. Gray boxes indicate the genes from this group that showed increased expression in a transgenic mouse line displaying Msi1 overexpression (OE) [21]. Msi1 targets previously identified by CLIP (Crosslinking and Immuno-precipitation) or RIP (Ribonuclear particle immuno-precipitation) [3,9,23] are labeled in red. (**C**) Western analysis of cell cycle and DNA replication genes affected by Msi1 KO in both glioblastoma (GBM) cell lines U251 and U343. The uncropped Western blots have been shown in Figure S4. (**D**) qRT-PCR analysis shows that transgenic expression of Msi1 in astrocytes increased the expression of cell cycle and DNA replication genes. Statistical significance calculated by multiple t-test. Data shown as means ±s.d. *** *p* < 0.001).

**Figure 2:**
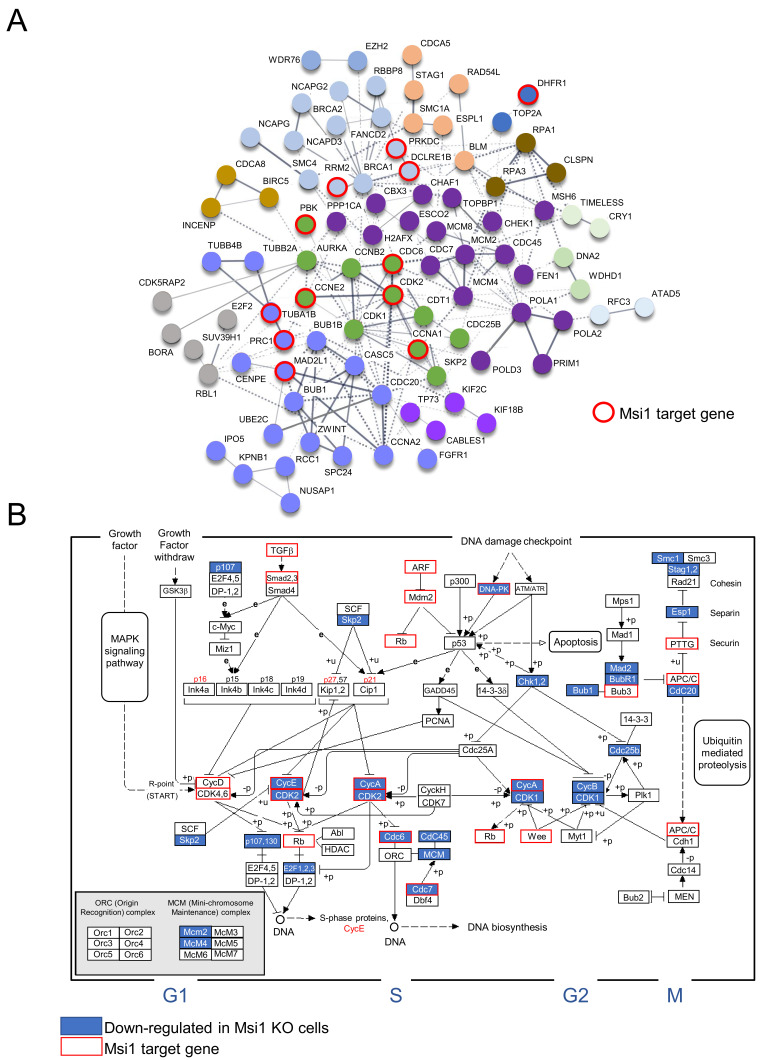
Msi1 impact on cell cycle and division. (**A**) Network shows cell cycle and/or division genes downregulated in Msi1 KO GBM cells in comparison to controls. The network was built using String [28] considering interaction (experimental evidence), text mining and co-occurrence. Different colors were used to indicate clusters of strongly related genes; Msi1 targets previously identified by CLIP or RIP [3,9,23] are labeled in red. (**B**) The impact of Msi1 KO on cell cycle is shown using KEGG’s cell cycle pathway. Downregulated genes in U251 and U343 Msi1 KO cells in comparison to respective controls are in blue boxes and Msi1 targets previously identified by CLIP or RIP [3,9,23] are labeled in red.

**Figure 3:**
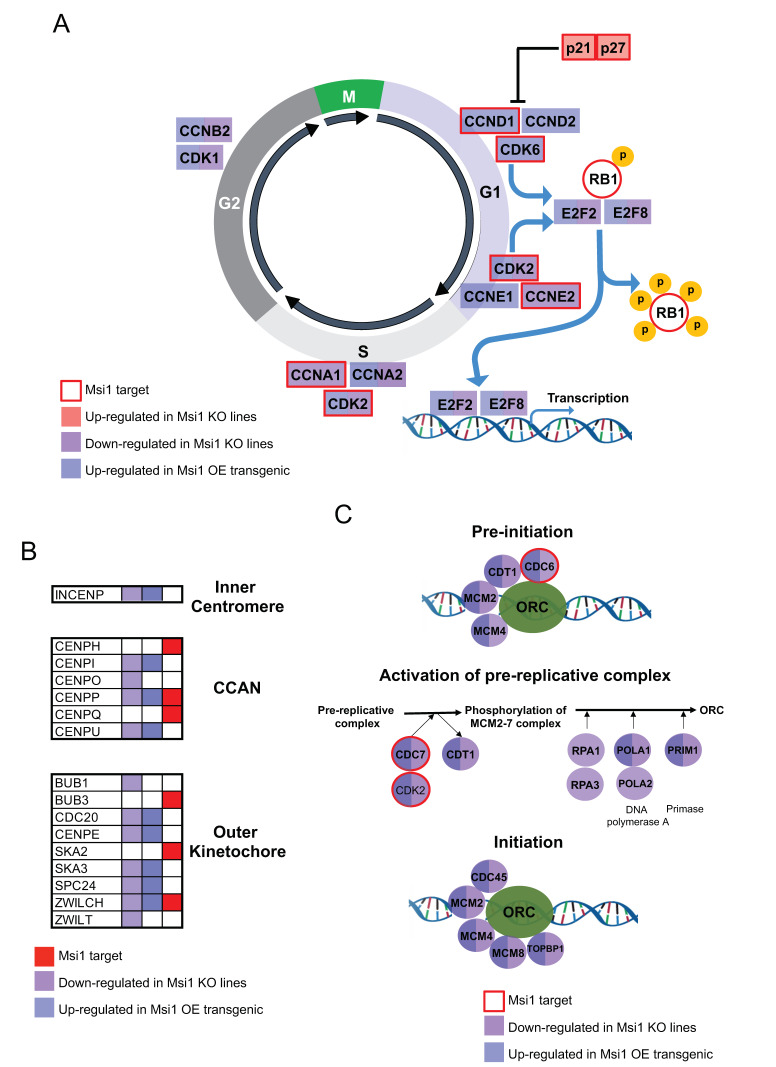
Musashi1 in cell cycle, cell division and DNA replication. (**A**) Cell cycle genes regulated by Msi1 according to different genomic analyses. (**B**) Important genes associated with the centromere that are regulated by Msi1 according to different genomic analyses. Centromere-associated network (CCAN). (**C**) Core DNA replication genes regulated by Msi1 according to different genomic analyses in the pre-initiation, activation of pre-replicative complex and initiation phases of DNA replication. Down-regulated genes in Msi1 KO cells in reference to controls are identified in purple, genes determined to be up-regulated in transgenic tissue with Msi1 OE [21] are identified in blue and Msi1 targets previously identified by CLIP or RIP [3,9,23] are labeled in red.

Msi1 is known to regulate genes associated with a variety of cellular processes and pathways [5,12]. To determine which ones are affected by Msi1 the most in GBM and define its core contributions to tumorigenesis, we conducted an integrated genomic study. Analysis of GBM U251 and U343 Msi1 KO cells and respective controls by RNA-seq identified a common set of ~300 downregulated genes strongly associated with cell cycle/division and DNA replication/repair (Figure 1, Figure 2 and Figure 3, Tables S1–S4). This list includes many Msi1 targets identified by CLIP (Crosslinking and Immuno-precipitation) or RIP (Ribonuclear particle immuno-precipitation) [3,9,23]. On the other hand, we have observed increased expression of two critical cell cycle regulators and Msi1 targets, p21 and p27 [24,25] (Figure 1, Figure 2 and Figure 3, Table S3). Supporting Msi1 impact on the cell cycle/division and DNA replication, we observed that several downregulated genes in Msi1 KO GBM cells linked to these processes showed increased expression in a murine transgenic line with Msi1 overexpression (OE) [21] (Figure 1B, Table S5). Moreover, these two gene sets show remarkable similarity in GO-enriched terms, especially concerning DNA replication and cell cycle/division (Figure 1A, Table S5). To determine if Msi1 could trigger the expression of cell cycle/division and DNA replication in neuronal cells, we transduced murine astrocytes with control and lentiviruses expressing Msi1. Astrocytes express very low levels of Msi1; in a qRT-PCR analysis, Msi1 could not be detected in the empty vector control group, while the Ct value reached 28 in astrocytes transduced with Msi1 expressing lentiviruses. The increased Msi1 levels obtained via lentiviral transfection induced the expression of cell cycle/division and DNA replication genes (Figure 1D). This result suggests that by turning on Msi1 expression in astrocytes, we could trigger cells to re-enter the cell cycle. Since Msi1 has been implicated in regulating cancer stem cells [5,12,26], we also investigated its impact on glioma stem cells (GSCs). Msi1 knockdown decreased the proliferation of GSCs 19NS and 84NS and also decreased the expression of cell cycle and DNA replication genes (Figure S1).

Analysis to identify associations between cell cycle/division genes affected by Msi1 KO in both GBM lines revealed a highly interconnected network (Figure 2A). To put the results into perspective, we mapped genes affected by Msi1 KO as well as previously identified Msi1 targets [3,9,23] to the KEGG cell cycle pathway (Figure 2B and Figure 3A). This analysis also established new regulatory functions for Msi1 by showing that several genes that form the centromeric complex (Figure 3B), as well as genes participating in different stages of DNA replication, are altered in Msi1 KO cells (Figure 3C).

**Figure 4:**
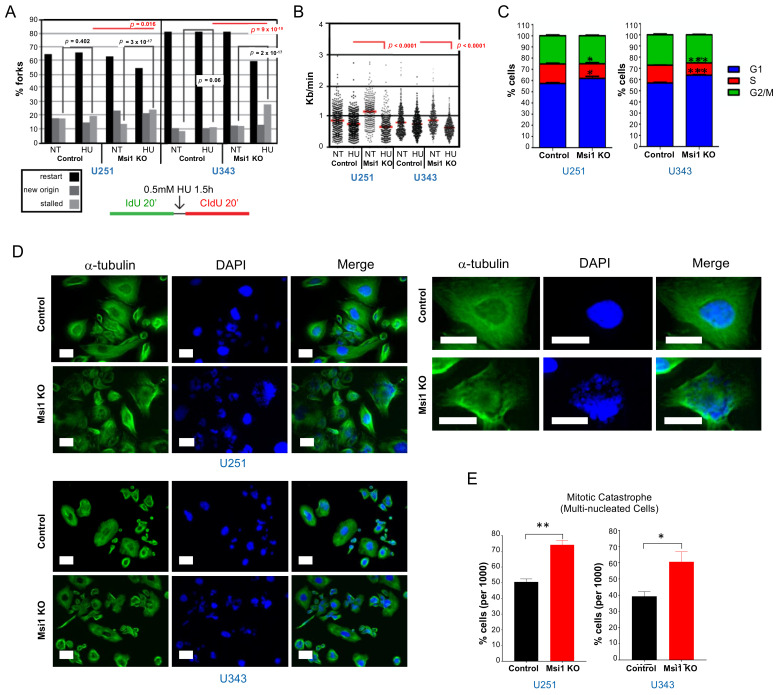
Musashi1 regulates DNA replication and cell cycle and division. (**A**) Graphs display the % of forks that are in restart, new orientation and stalled in Msi1 KO vs. control GBM cells. Statistics are shown for only stalled. The comparisons between no treatment (NT) and HU are shown in black. The comparisons between controls and Msi KO clones after HU treatment are shown in red. Statistical significance was calculated by Chi-square with Yate’s correction and Fisher’s exact test (ns: not significant). (**B**) The graph shows replication fork (RF) speed. The comparisons between controls and Msi1 KO cells after HU treatment are shown in red. Statistical significance calculated by Mann-Whitney test. The mean values are represented as horizontal red lines. RF speed of Msi1 KO clones showed a slowdown. (**C**) U251 and U343 Msi1 KO cells displayed changes in cell cycle progression (accumulation of cells in G1) in comparison to respective controls. (**D**) The aspect of U251 and U343 Msi1 KO and control cells exposed to Paclitaxel. On the left, staining with anti-α-tubulin; on the right, staining with DAPI shows an increased number of multinucleated cells in Msi1 KO cells. Scale bar = 10 µm. (**E**) The graph shows the number of multinucleated cells in U251 and U343 control and Msi1 KO cells after treatment with Paclitaxel (* *p* < 0.05, ** *p* < 0.01, *** *p* < 0.001).

To link changes in gene expression triggered by Msi1 KO to phenotypes, we conducted assays to measure alterations in the cell cycle, cell division, and DNA replication of glioblastoma cells. To evaluate Msi1’s impact on DNA replication, DNA fiber analysis was used to quantify stalled replication forks in response to a mild dose of hydroxyurea (HU) [29] and replication speed. U251 and U343 Msi1 KO cells and respective controls were pulse-labeled with ldU (green), followed by HU. After HU removal, cells were pulse-labeled with CIdU (red) [30]. Fibers labeled with only ldU (green) were counted as stalled RFs. HU inhibits ribonucleotide reductase [31] and impairs the restart of replication forks [32]. This dose of HU does not cause breaks [32,33] and has a mild effect on replication fork restart and origin firing in control cells. We found that Msi1 KO cells show significantly more stalled forks than controls; similarly, replication fork speed was compromised (Figure 4A,B).

We determined by flow cytometry changes in cell cycle distribution taking place in Msi1 KO U251 and U343 cells in relation to respective controls. In agreement with our previous observations [3,9,21,23], we detected a decrease in the number of cells in the S phase and a concomitant increase in G1 (Figure 4C). In fact, several Msi1 targets in the cell cycle are important players in G1: CDK2, CDK6, CCNA1, CCNE2, p21, and p27 (Figure 3). To evaluate Msi1 participation in cell division, U251 and U343 Msi1 KO cells and respective controls were exposed to 20nM of Paclitaxel for 24 h and later stained with α-tubulin and DAPI. In both cases, Msi1 KO cells showed an increased number or polynucleated cells, reflecting mitotic catastrophe (Figure 4D,E). Interestingly, we have recently described an antagonistic model between Msi1 and miR-137 [4]. A comparison between the set of genes downregulated in Msi1 KO cells and miR-137 targets showed that a large number of cell cycle/division genes affected by Msi1 are also regulated by miR-137 (Table S3).

Genes upregulated in Msi1 KO cells are strongly associated with cell differentiation and development (Figure S2, Table S4). This result is in agreement with Msi1 role in maintaining the stem cell state and preventing neuronal differentiation [4,34,35,36].

### 2.2. Glioblastoma Msi1 KO Cells Are More Sensitive to DNA Replication and Cell Cycle Inhibitors

**Figure 5:**
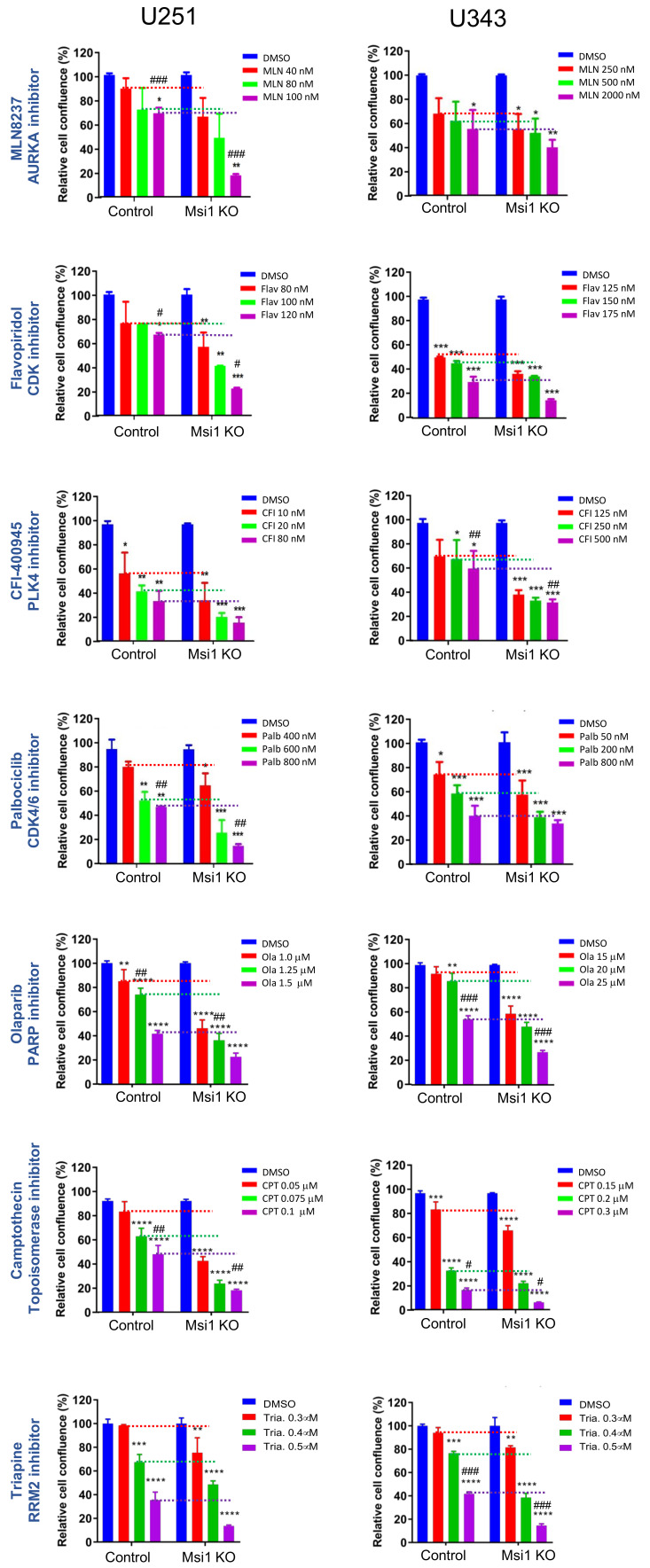
GBM cells with Msi1 KO are more sensitive to cell cycle and DNA replication inhibitors. Graphs show the impact of different cell cycle and DNA replication inhibitors on proliferation (72 h) of U251 and U343 Msi1 KO cells in reference to their respective controls (DMSO treated). Statistical significance was calculated by one-way ANOVA and t-test. Data shown as means ± s.d. (* *p* < 0.05, ** *p* < 0.01, *** *p* < 0.001, **** *p* < 0.0001). Synergy between “Msi1 KO” and cell cycle and DNA replication inhibitors was determined by the statistical response additivity approach [41], with # indicating the drug concentration in which we observed the statistically most significant synergy (# < 0.05, ## < 0.01, ### < 0.001).

In cases of highly aggressive tumor types like GBM, drug combinations are often the most effective strategy. Msi1 inhibition has been proposed as a treatment option against different malignancies and we and others have developed inhibitors against Msi1 [7,37,38,39,40]. We tested if Msi1 KO lines show increased sensitivity to cell cycle/DNA replication inhibitors in respect to controls. U251 and U343 Msi1 KO and control lines were treated with increasing concentrations of six different inhibitors (MLN8237, AURKA inhibitor; Flavopiridol, CDK inhibitor; Olaparib, PARP inhibitor; CFI-400945, PLK4 inhibitor; Camptothecin, Topoisomerase inhibitor; and Triapin, RRM2 inhibitor) and their impact on cell proliferation was assessed using the Incucyte system. In each case, control and Msi1 KO lines were normalized against respective DMSO-treated cells. This allowed us to directly compare control and Msi1 KO lines at each concentration. Overall, results indicated that Msi1 KO conferred cell sensitivity to cell cycle and DNA replication inhibitors (Figure 5). We evaluated results from a different perspective and calculated the synergy between “Msi1 KO” and cell cycle and DNA replication inhibitors using the statistical response additivity approach [40]. In each experiment, we indicate the drug concentration in which we observed the most significant interaction (Figure 5). Our data support the idea of concomitant Msi1 and DNA replication/cell cycle inhibition as a potential therapeutic strategy to treat GBM. In this respect, we have previously observed synergistic effects when Msi1 inhibitor Luteolin was combined with radiation or Olaparib to treat glioblastoma cells [7]. We expanded this analysis and determined the value of combining Camptothecin and Luteolin to treat glioblastoma cells. We used a single dose of Luteolin and increasing concentrations of Camptothecin and measured the impact on U251 cell proliferation. Results suggest synergism between these two drugs (Figure S3).

### 2.3. E2F2 and E2F8 are Mediators of Msi1 Impact on DNA Replication and Cell Cycle/Division

**Figure 6:**
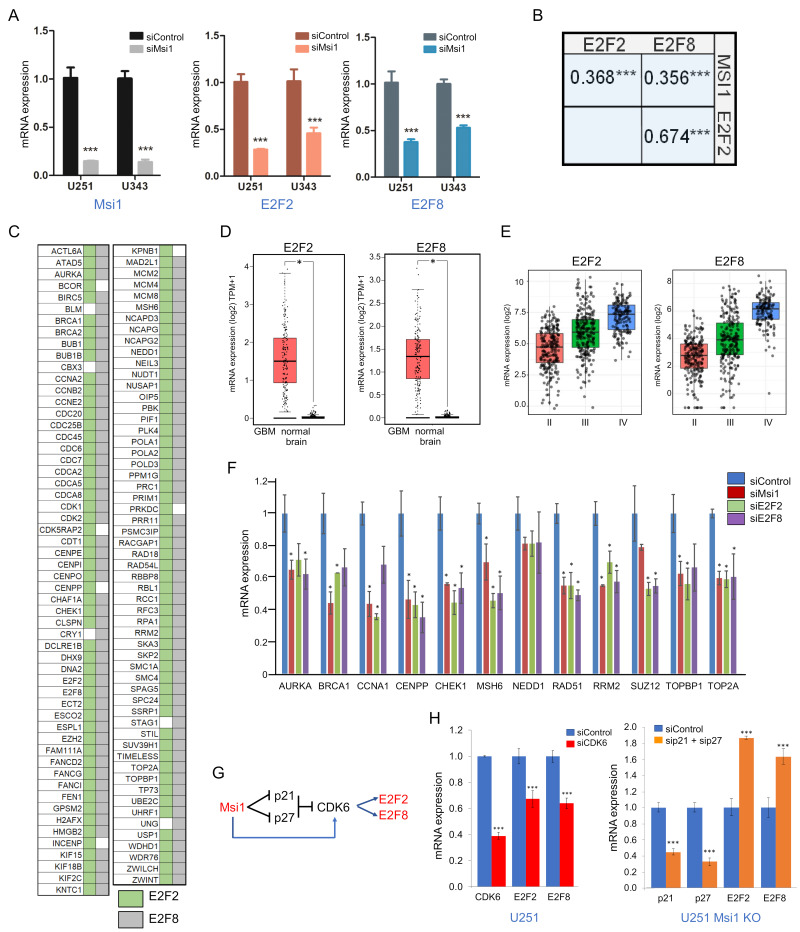
E2F2 and E2F8 are mediators of Msi1 impact on cell cycle and DNA replication genes. (**A**) qRT-PCR data showing that E2F2 and E2F8 decreased expression upon Msi1 knockdown in U251 and U343 cells. Statistical significance calculated by multiple *t*-test (*** *p* < 0.001). (**B**) Expression correlation (R values) between Msi1, E2F2 and E2F8 in TCGA GBM samples (*** *p* < 0.001). (**C**) The table shows genes affected by Msi1 KO in U251 and U343 cells that also display high expression correlation with E2F2 and E2F8 in GBM samples. Pearson correlation, all genes *** *p* < 0.001, R > 0.3. (**D**) E2F2 and E2F8 mRNA expression levels in normal brain vs. glioblastoma according to GEPIA [54]. (**E**) E2F2 and E2F8 mRNA expression in gliomas grades II, II and IV (TCGA samples). Data shown in B, C and E was generated with Gliovis [55]. (**F**) qRT-PCR data shows the impact of Msi1, E2F2 and E2F8 knockdown on the expression of the cell cycle, cell division and DNA replication genes in U251 cells. (**G**) Model showing that Msi1 regulates the expression E2F2 and E2F8 via CDK6, p21 and p27. (**H**) qRT-PCR data shows the impact of CDK6, p21 and p27 knockdown on the expression of E2F2 and E2F8. Left panel, CDK6 KD was performed in U251 cells. Right panel, p21 and p27 double KD were performed in U251 Msi1 KO cells. Statistical significance calculated by multiple t-test. Data shown as means ± s.d. (* *p* < 0.05, *** *p* < 0.001).

We checked downregulated genes in Msi1 KO cells to determine which ones were previously identified as Msi1 targets by CLIP and RIP [3,9,23]. Most of these genes do not display Msi1 binding sites in their transcripts and therefore, we conclude that they are very likely indirectly regulated by Msi1. To identify Msi1 mediators, meaning regulators that ultimately drive the expression of these genes, we ran GSEA (gene set enrichment analysis) using Enrichr [42] and MsigDB [43]. Both analyses identified matches with the E2F family of transcription factors. Among members of this family, E2F2 and E2F8 emerged as the strongest candidates. E2F2 and E2F8 displayed reduced expression in U251 and U343 Msi1 KO lines while their expression levels increased in Msi1 OE mouse tissue [21,44,45] (Figure 1B,C, Tables S3 and S5). The impact of Msi1 on the expression of E2F2 and E2F8 was corroborated via Msi1 knockdown in U251 and U343 cells (Figure 6A). Moreover, Msi1, E2F2, and E2F8 show high expression correlation in GBM samples from the TCGA (Figure 6B). A high expression correlation was also observed between E2F2/8 and a large number of genes downregulated in Msi1 KO cells (Figure 6C, Table S6).

E2F2 and E2F8 have been implicated in GBM development, cell cycle and DNA replication [46,47,48,49,50] and were identified as hits in a Temozolomide sensitivity screening [51]. We observed that E2F2 and E2F8 mRNA levels are higher in GBM when compared to normal brain and gliomas grade II and III (Figure 6D,E) and, similarly to Msi1, their expression levels decrease during neuronal differentiation [4].

To further support the role of E2F2 and E2F8 as mediators of Msi1’s impact on cell cycle/division and DNA replication genes, we conducted RNA-seq experiments in U251 control vs. E2F2 or E2F8 knockdown cells. The results were then compared to the ones obtained in the U251 Msi1 KO vs. control study. Changes in the expression of a relevant set of genes altered upon Msi1, E2F2 and E2F8 KO/KD were validated by qRT-PCR (Figure 6F). Gene ontology analysis of the 605 genes downregulated both in U251 Msi1 KO and E2F2 and/or E2F8 knockdown cells shows cell cycle, DNA replication, and cell division as top enriched terms (Table S7). Next, to find direct evidence of regulation, we searched the ENCODE ChIP-seq database. We found datasets for E2F8 and looked for evidence of E2F8 binding to genes down-regulated after its knockdown in U251 cells. The 305 genes identified show a dramatic enrichment for processes linked to DNA replication and cell cycle regulation (Table S8). Finally, single-cell analysis of glioblastoma tumors identified a cell cluster showing increased expression of cell cycle-related genes including E2F8 and several genes downregulated in U251/U343Msi1 KO cells [52] (Table S8).

Although E2F2 and E2F8 appear to function as Msi1 main mediators in cell cycle/division and DNA replication, previous CLIP and RIP analyses [3,9,23] did not identify these two genes as direct Msi1 targets. Based on what is known regarding the regulation of E2F transcription factors [50], we decided to test if the previously identified Msi1 direct targets CDK6, CDKN1A/p21, CDKN1B/p27 [3,21,24,53] affect E2F2 and E2F8 levels in glioblastoma cells. Msi1 functions as a repressor of p21 and p27 but as a positive regulator of CDK6 expression (Figure 6G). In glioblastoma Msi1 KO cells, we observed increased p21 and p27 expression (Table S3). We then performed a double knockdown of p21 and p27 in U251 Msi1 KO cells and observed as predicted, up-regulation of E2F2 and E2F8 (Figure 6H). On the other hand, knockdown of CDK6 in U251 cells reduced the expression of E2F2 and E2F8 (Figure 6H). Therefore, we suggest that activation of E2F2 and E2F8 expression by Msi1 involves blockage of p21 and p27 and activation of CDK6.

## 3. Discussion

### 3.1. Musashi1 Modulates Cell Cycle Progression and DNA Replication in GBM Cells

Regulation of cell cycle progression is crucial for genome integrity maintenance. Cells are particularly sensitive during S phase, when the genome is replicated through a fundamental process that requires spatio-temporal coordination of many replication origins and activation of checkpoint cascades that impose cell-cycle arrest if necessary, thus preventing the propagation of damaged DNA [56]. Expanding on Msi1 function in cell cycle progression [3,26,57,58,59,60,61,62], we showed that Msi1 levels influence the expression of genes implicated in different stages of the cell cycle. In Msi1 KO glioblastoma cells, we observed that G1-S transition is disrupted, agreeing with an increase in expression of Msi1 targets p21 and p27 and downregulation of CDK2 and CCNE2. p21 and p27 accumulation inhibits the activity of the cyclin complexes and prevents the transition from G1 to S phase. In fact, both CDK2 and CCNE2 are inhibited by p21 and p27. CDK2 plays a crucial role in regulating several events in cell cycle/division including centrosome duplication, G1-S transition, DNA synthesis, and G2 progression [63]. CCNE2 is a G1 cyclin that interacts with CDK2 [63]. CCNE2 activity is regulated in a cell cycle-dependent manner, having its peak activity at the G1/S transition. CCNE2 ectopic expression accelerates G1 in human cells [62]. Both CDK2 and CCNE2 are highly expressed in GBM and linked to poor patient survival [1,64].

Regulation of DNA replication was identified as a new core function of Msi1 in glioblastoma cells. Msi1 KO cells showed decreased expression of several genes participating in the different stages of DNA replication, including three direct targets of Msi1 (CDC6, CDC7 and CDK2) [3]. Replication begins at specific positions in chromosomes where a protein complex, five subunits of ORC (origin recognition complex) and CDC6, bind to DNA. CDT1 brings the MCM2-7 helicases to the ORC-CDC6 complex and DNA replication forks are initiated. Msi1 levels influenced the expression of MCM2, MCM4, CDC6 and CDT1 [21]. CDC6 is highly expressed in high-grade gliomas and was significantly associated with decreased patient survival [64,65,66,67]. CDC6 was also identified as part of 20 genes that is consistently expressed in GSC cultures but not expressed in NSC cultures [67]. CDC7 is an evolutionarily conserved serine-threonine kinase that regulates G1/S phase transition and DNA replication [68]. CDC7 expression is high in GBMs, it is required for tumor proliferation, implicated in radio-resistance, invasion and tumor growth [69,70].

We also determined that Msi1 levels modulate the expression of RRM2, a ribonucleotide reductase, required in cell replication and DNA synthesis [66,71,72]. It was recently shown that BRCA1, which is also affected by Msi1 levels, promotes the transcription of RRM2 [56]. Gliomas exhibit pronounced genomic instability and develop a dependence on BRCA1. Both BRCA1 and RRM2 were determined to be negative prognostic factors for glioma patient survival and implicated in tumor development [56,73]. Treatment with Triapine, an RRM2 specific inhibitor, impaired growth of GBM cells and sensitized GBMs to PARP inhibitor Olaparib. Interestingly, we determined that Msi1 KO cells are more sensitive to Triapine and Olaparib and a synergistic effect was observed when we treated glioblastoma cells with Olaparib and Luteolin, a Msi1 inhibitor [7].

### 3.2. Musashi1 Regulates the Expression of Members of the Centromeric Complex

Accurate chromosome segregation in mitosis maintains genome stability. Chromosome segregation depends on the attachment of spindle microtubules to chromosomal centromeres, through kinetochore protein complexes. The kinetochore is represented by the centromeric chromatin-binding inner kinetochore composed of the 16-subunit constitutive centromere-associated network (CCAN) and the microtubule-binding outer kinetochore that contains 10-subunit Knl1–Mis12–Ndc80 (KMN) network, which links the inner kinetochore to microtubules. Deregulation of kinetochore-microtubule attachments has been implicated in driving chromosome instability and cancer evolution [74]. Msi1 regulates the expression of several components of the inner and outer kinetochore and as a possible consequence of this regulation, Msi1 KO cells displayed increased mitotic catastrophe and changes in cell cycle distribution.

The budding uninhibited by Benzimidazole (BUB) and the mitotic arrest deficient (MAD) gene families are responsible for mitotic spindle assembly checkpoint (SAC), ensuring the fidelity of chromosome segregation by recruiting members of the mitotic checkpoint complex and activating the spindle checkpoint [75]. GBMs tend to show an increase in the expression of mitotic spindle checkpoint genes [76]. We established that Msi1 levels affect the expression of Bub1, Bub1B, MADL1 and CDC20 and Bub3 is a direct target of Msi1 in glioblastoma cells [3]. Bub1B was identified as top-hit in a genomic screening to identify kinases central to the viability of GSCs but dispensable to proliferating NSCs [77]. Bub1 plays an important role in GBM proliferation and radio-resistance [75,78]. Bub3 is required to recruit both Bub1 and BubR1 to kinetochore [79], and its inhibition resulted in chromosome segregation defects through the loss of the Bub1 function at kinetochore-microtubule attachments [80].

The SAC proteins prevent activation of the ubiquitin ligase anaphase-promoting complex/cyclosome (APC/C) by targeting APC/C’s cofactor, CDC20. Following correct chromosome alignment and tension at the kinetochore, Cdc20 inhibition is released to activate the APC/C via dissociation of MAD2, and anaphase is initiated [81]. By targeting MAD2L2, Msi1 would ensure accurate mitosis by preventing premature activation of APC during prometaphase and early anaphase. It has been shown that loss of MAD2L2 results in dysregulation of APC/C and unscheduled mitotic exit [82]. Msi1 levels affected the expression of the oncogene CDC20 [83,84]. CDC20 increased expression has been observed in high-grade gliomas and linked to poor patient survival [76,85,86,87] while its knockdown reduced invasion, self-renewal, proliferation of GSCs and impaired tumor initiation [86,88,89].

### 3.3. E2F2 and E2F8 Are Critical Mediators of Msi1 Impact on Cell Cycle and DNA Replication

The transcription factors E2F2 and E2F8 were identified as important mediators of Msi1 function in cell cycle/division and DNA replication. The E2F transcription factors have been defined as central expression regulators of cell cycle genes [50]. They play a central role in regulating G1/S transition and progression through S phase, promoting cellular transformation [47]. The family has eight members that function as activators or repressors depending on the context and regulate one another. E2Fs show increased expression in many tumors as a result of enhanced cyclin-dependent kinases (CDKs) activity or, inactivation of CDK inhibitors or RB Transcriptional Corepressor 1 (RB1). Alterations in E2F genes have been linked to poor patient survival and can induce tumor formation in mice [47,48,49,50]. A bioinformatic study defined the E2F family as the main contributor to the expression of up-regulated genes in glioblastoma [90]. In a recent study to evaluate early changes in expression in glioblastoma cells after radiation, we determined that down-regulation of genes implicated in the cell cycle, DNA repair and DNA replication is likely the result of decreased expression of FOXM1, E2F1, E2F2 and E2F8 [91].

Analysis of high-grade glioma (HGG) indicated that E2F2 and E2F8 are highly correlated with oncogenes driving proliferation and therapeutic resistance and E2F8 independently predicts poorer survival [92]. E2F8 and E2F2 have been also implicated in the maintenance of glioma stem cell phenotypes [90,91] and cell transformation [93,94,95]. E2F2 is up-regulated in CD133(+) astrocytoma cells and was implicated in astrocyte transformation [48]. Another study showed that E2F2 is required for GSC self-renewal and in this context, it regulates the transcription of members of the inhibitor of differentiation (ID) helix-loop-helix gene family [93]. Similarly, to what we observed in Msi1 knockdown or KO cells [3], inhibition of E2F2 expression arrests cell division in the G1 phase [94]. E2F8 is still poorly characterized in the context of brain tumors but it has been implicated in the development of various cancer types including lung cancer, lymphoma, colon cancer, cholangiocarcinoma, prostate cancer, hepatocellular carcinoma and breast cancer [96,97,98,99,100,101,102,103,104,105]. E2F8 high expression is strongly associated with a worse outcome of GBM patients and radio-resistance while E2F8 knockdown decreased proliferation and tumor growth and inhibited invasion, migration and the expression of genes implicated in metastasis [49]. Bioinformatics analysis established a link between E2F8 and multiple oncogenic pathways, including cell cycle, DNA repair, STAT3, TGFRβ and WNT [105].

E2F family members emerge as potential therapeutic targets. However, developing inhibitors against transcription factors remains very challenging. An alternative is to identify upstream regulators. Integrated ChIP-seq and RNA-seq analyses E2Fs are the primary downstream targets of that Bromodomain and extraterminal (BET) proteins in both GBM cell lines and patient-derived GBM spheres [106]. Several BET bromodomain inhibitors (BBIs) are available and being evaluated as anti-cancer agents [107,108,109,110].

### 3.4. Targeting RNA-Binding Proteins in Cancer Therapy

A growing number of RBPs has been implicated in tumor initiation and development and they have started to be explored as targets in cancer therapy [111,112]. Diverse strategies have been employed to target oncogenic RBPs. Small molecule inhibitors have been developed, especially in the case of splicing regulators [113]. Another strategy employs RNA aptamers or modified RNA oligos containing the binding motif of the RBP to be targeted. This strategy often requires oligo optimization and the inclusion of RNA modifications to increase stability and delivery [114]. A different approach is to target the RBP expression levels. For instance, an antisense oligonucleotide against eIF4E delivered by intravenous injections prevented tumor growth by repressing the translation of oncogenic factors dependent on elF4E function [115].

A single agent is unlikely to be effective in treating high aggressive tumors like glioblastoma. We and others have developed inhibitors against Musashi proteins [7,35,36,37,38,116,117,118]. We have identified a Msi1 inhibitor, luteolin, which showed synergistic interactions with radiation, Olaparib and Camptothecin. Based on our results indicating that Msi1 KO cells are more sensitive to cell cycle and DNA replication inhibitors, other agents in combination with Msi1 inhibitor could be explored to treat glioblastoma.

## 4. Materials and Methods

### 4.1. Cell Lines

Glioblastoma cell lines U251 and U343 were obtained from Uppsala University (Uppsala, Sweden). Msi1 CRISPR KO lines were developed two for each cell type. To prepare Msi1 KO U251 and U343 lines, two Msi1 target sequences (5′-CACCGTGGGGCGCGTCAGTCTCCAT-3′/5′-CACCGCGAATACTTCGGCCAGTTCG-3′) were cloned into lenti–clustered regularly interspaced short palindromic repeat v2 plasmid (Addgene, Cambridge, MA, USA; Cat# 52961;) and used for co-transfecting U251 and U343 cells. After selection using puromycin, cells were submitted to cell cloning by serial dilution in 96-well plates. Single colonies were transferred to a 12-well plate and allowed to grow [4,8]. All cells were cultured in DMEM medium (HyClone, Logan, UT, USA; Cat# SH30243.01) supplemented with 10% FBS (Corning, NewYork, NY, USA; Cat# 35015CV) and 1% penicillin/streptomycin (Gibco, Grand Island, NY, USA; Cat# 10378016). Cells were harvested by using trypsin, counted with the Countess automated cell counter (Invitrogen, Bedford, MA, USA) using trypan blue and re-plated in tissue culture plates for transfection and further assays. GSC lines (19NS and 84NS) [119] were cultured in serum-free media consisting of Neurobasal-A media supplemented with B-27, Sodium Pyruvate, Glutamax, Pen/Strep, 20ng/mL EGF (ThermoFisher Scientific, Grand Island, NY, USA), and 20 ng/mL hFGF (PeproTech, Rocky Hill, NJ, USA).

### 4.2. Cell Transfection and siRNA Knockdown

GBM cells were transiently transfected with small interfering RNA (siRNA) or control siRNAs using Lipofectamine RNAiMAX (Invitrogen, Carlsbad, CA, USA Cat# 13778150) and harvested 72 h for different assays. siRNAs were obtained from Dharmacon (Msi1, Cat# L-011338-00) or Ambion (E2F2 Cat# S4408, E2F8 Cat# S36212, p21 Cat# 118565, p27 Cat# 118712, CDK6 Cat# 103566). ON-TARGET plus Non-targeting siRNA (Dharmacon, Lafayette, CO, USA; Cat# D-001810-01-05) was used as a control. Knockdown levels of the target RNA compared to the control siRNA transfection were measured by quantitative Real-Time PCR (qRT-PCR). GSC lines were dissociated with Accutase (ThermoFisher) and reversibly transfected at a density of 10^4^ cells/well with siRNA control or siRNA Msi1 and plated in 96-well plates pre-coated 3 h prior with Geltrex™ LDEV-Free Reduced Growth Factor Basement Membrane Matrix (19.2–28.8 μg/mL).

### 4.3. Astrocyte Cell Culture and Lentivirus Infection

Murine astrocytes were cultured in DMEM-F12 media with 10% FBS. 105 cells/well in 12-well plated were transduced with lentiviral particles (GeneCopoeia, product ID: U0081) expressing Msi1 or empty vector control (MOI of 5) in the presence of 5 μg/mL of polybrene. Total RNA was extracted for analysis three days after infection.

### 4.4. RNA Extraction and qRT-PCR Analysis

Total RNA was extracted from cells using TRIzolTM reagent (Thermo Fisher Scientific, Grand Island, NY, USA; Cat# 15596018), following the manufacturer’s instruction. cDNA was synthesized from 2000 ng of total RNA using High-Capacity cDNA Reverse Transcription Kit (Thermo Fisher Scientific; Cat# 4368814). Quantitative PCR was performed using TaqMan Universal PCR Master Mix (Applied Biosystems, Foster City, CA, USA) or PowerUp SYBR Green Master Mix (Thermo Fisher) and reactions were performed on ViiA™ 7 Real-Time PCR System (Applied Biosystems). Data were acquired using ViiA 7 RUO software (Applied Biosystems) and analyzed using the 2−ΔΔCT method with GAPDH as an endogenous control. Probes and primers used in qRT-PCR are listed in Table S9.

### 4.5. RNA-Seq Analysis

We performed RNAseq (50 nt single reads) of the poly-A fraction using Illumina protocol. Sequencing adapters were first trimmed using cutadapt [120] with a minimum quality threshold set to 5 (-u 5) with remaining parameters as default. The RNA-seq samples were mapped to the human transcriptome (build GRCh38; GENCODE [121] GTF (v25)) using STAR [122] by allowing at most two mismatches (-outFilterMismatchNmax 2). Only uniquely mapping reads were retained (-outFilterMultimapNmax 1). Reads were quantified using feature counts [123]. Differentially expressed genes were identified using DESeq2 [124] with the covariates being the knockdown status (knockdown vs. control). A list of processed read counts and raw sequencing data have been deposited to GEO (accession GSE151155).

### 4.6. Gene Ontology and Network Analyses

Gene Ontology and pathway enrichment analyses of down and up-regulated gene sets were performed using Panther [125] and ShinyGo [27]. For both analyses, we considered terms to be significant if *p*-values were <0.05 and fold enrichment was >2.0 (adjusted for false discovery rates). We used the STRING database (v11) [28] to construct protein-protein interaction and determined associations among genes in a given dataset. The interactions were based on experimental evidence procured from high-throughput experiments, text-mining, and co-occurrence (interaction score 0.40).

### 4.7. Expression Correlation and Expression Analyses

We performed expression correlation analyses to identify genes with a strong positive correlation with E2F2 and E2F8 in the TCGA glioblastoma set (RNA-seq samples) (R ≥ 0.3, *p*-value 0.05) and used Pearson correlation. The analysis was done using resources in Gliovis [55]. The list of genes downregulated in U251 and U343 Msi1 KO cells was compared to the list of genes upregulated in a Msi1 OE transgenic line described in [21].

### 4.8. Western Blot

Cell pellets were re-suspended and sonicated in the Laemmli sample buffer. Extracted proteins were separated on SDS-PAGE gel and transferred to PVDF membranes. Membranes were blocked in TBS-T+5% milk and then probed with the following antibodies: Msi1 (Abcam, Cambridge, MA, USA; ab52865), RRM2 (GeneTex, Irvine, CA, USA GTX103193), AURKA (Cell Signaling, 14475S), CDK2 (Cell Signaling, 2546S), CDC6 (Cell Signaling, 3387S), E2F8 (GeneTex, GTX 112299) and β-tubulin (Sigma-Aldrich, St Louis, MO, USA). Horseradish peroxidase (HRP)-conjugated goat anti-rabbit antibody (Santa Cruz Biotechnology, Santa Cruz, CA, USA) or HRP-conjugated goat anti-mouse antibody (ThermoFisher) was used as a secondary antibody. Bound antibodies were detected using Immobilon Western Chemiluminescent HRP Substrate (Millipore, Billerica, MA, USA).

### 4.9. Cell Cycle Analysis

Analysis of the cell cycle was performed by flow cytometry after Propidium Iodide (PI) staining. Control and Msi1 KO U251/U343 cells were plated into six well plates (6 × 10^4^ cells/well for U251 and 1.2 × 10^5^ for U343) and fetal bovine serum (FBS) was removed the following day from the medium to subject the cells to synchronization for 24 h. The cells were further harvested, washed with cold PBS twice and fixed with 70% cold ethanol solution and then kept at −20 °C overnight. This was followed by the centrifugation (400× *g* at 4 °C for 5 min) of the cells and the resulting cell pellet was resuspended in PBS with RNAse A (10 ng/mL) and incubated at 37 °C for 30 min. Then, the cells were centrifuged and added PBS with PI at a final concentration of 50 μg/mL for staining. The cells were incubated and analyzed for cell cycle using FACS BD caliber. 19NS and 84NS GSCs were dissociated with accutase and reversibly-transfected at a density of 3 × 10^5^ cells/well with 20 nM of siRNA control or siRNA Msi1 and plated in 6-well plate. Four days later, cell cycle analysis was performed as above. All experiments were performed in triplicate.

### 4.10. Cell Division Analysis

U251 and U343 control and Msi1 KO cells were plated in 96 wells at a density of 8 × 10^4^. Cells were synchronized by serum starvation for 24 h. Following synchronization, cells were exposed to 20 nM of Paclitaxel for 24 h. Cells were fixed in 4% PFA and stained with Alpha-Tubulin and DAPI. The experiment was performed in triplicates and 1000 cells were counted per replicate. Differences were assessed with a student’s *t*-test. *p* < 0.01.

### 4.11. Cell Proliferation Assay

Control and Msi1 KO U251/U343 cells, as well as control and control and RRM2 knockdown U251/U343 cells, were plated onto 96-well plates (800 cells/well for U251 and 1600 for U343). After 24 h the cells were treated with the different inhibitors or DMSO. Plates were transferred to the IncuCyte automated microscope system (Essen BioScience, Ann Arbor, MI, USA) and cells were counted every 2 h for 5–7 days. All experiments were performed in triplicate.

### 4.12. DNA Replication

The procedure was performed as described previously [126]. At least 500 fibers were counted per condition. The lengths of IdU and CldU patches were measured using Zen 2.3 software (Zeiss, Jena, Germany) and micrometer values were converted into kb using conversion factor 1 μm = 2.59 kb [127].

### 4.13. Impact of Cell Cycle and DNA Replication Inhibitors on Msi1 KO Cells

A stock solution of the different drugs was prepared at a final concentration of 10 mM in DMSO (Sigma Cat# D8418) and stored in aliquots at −20 °C. Aurora-A kinase inhibitor (MLN8237 Cat# 13602), Polo-like kinase 4 inhibitor (CFI-400945 Cat# 16850) and Cyclin-dependent kinases inhibitor (Flavopiridol Cat# 26024) were purchased from Cayman Chemical (Ann Arbor, MI, USA). CDK4 and CDK6 inhibitor (Palbociclib Cat# HY-50767) was purchased from MedChemExpress (Monmouth Junction, NJ, USA). Triapin (Cat# S7470), Olaparib (Cat# S1060), Camptothecin (Cat# S1288) were purchased from Sellectchem (Houston, TX, USA). Each assay was performed in triplicate. Analysis of drug synergism was performed in U251 cells. Single and combined treatment of Luteolin (Sigma Aldrich Cat# L9283) and Camptothecin (Cayman Chemical Cat# S1288) or Polo-like kinase 4 inhibitor (Cayman Chemical CFI-400945 Cat# 16850) were carried out. U251 cells were plated at 2000 cells/well in a 96-well plate. 24 h later, drugs were added and cell proliferation was recorded using Incucyte^®^ system. Each assay was performed in triplicate.

### 4.14. Analysis of Drug Synergism

U251 cells were plated onto 96-well plates (1500 cells/well). 24 h later, cells were treated with a low concentration of luteolin or DMSO plus different concentrations of Camptothecin. Plates were transferred to the IncuCyte and cells were counted every 4 h for 4–6 days. The synergistic effect of Luteolin and Camptothecin was determined via the combination index (CI) [128]. CI = AB/ (A × B) where: AB = measured value for combined treatment/value for the control (DMSO), A and B = value for the single treatment/value for the control. Thus, CI <1, =1 or >1, indicates that the combination treatments are synergistic, additive or antagonistic.

### 4.15. Statistical Analysis

Prism software (GraphPad Prism 7.0 - GraphPad Software, San Diego, CA, USA), one-way ANOVA, two-way ANOVA, *t*-test, Mann-Whitney test, Chi-square with Yates’ correction and Fisher’s exact test were used to analyze the data. A threshold of *p* < 0.05 was defined as statistically significant. To evaluate the synergy between “Msi1 KO” and cell cycle and DNA replication inhibitors, we used the statistical response additivity approach proposed by Slinker (1998) [41]. To account for multiple comparisons, we corrected the p-values using Bonferroni correction.

## 5. Conclusions

We established via a comprehensive genomic analysis that regulation of cell cycle/division and DNA replication are Msi1 core regulatory functions in GBM. Msi1 controls this gene network via two mechanisms: direct interaction and indirect regulation mediated by the transcription factors E2F2 and E2F8. Importantly, since glioblastoma lines with Msi1 KO displayed increased sensitivity to cell cycle and DNA replication inhibitors, we propose that a drug combination strategy (Msi1 + cell cycle/DNA replication inhibitors) could be a viable treatment option for GBM patients.

## Data Availability

The data presented in this study are available in the Supplementary Materials. A list of processed read counts and raw sequencing data have been deposited to GEO (accession GSE151155).

## Supplementary Materials

The following are available online at https://www.mdpi.com/2072-6694/13/7/1494/s1, Figure S1: Msi1 knockdown suppressed GSC lines proliferation and affected the expression of cell cycle and DNA 12 replication genes, Figure S2: Glioblastoma cells with Musashi1 knockout show increased expression of a network of genes implicated 16 in development and cell differentiation, Figure S3: Synergistic effect of Camptothecin(CPT) and Luteolin (Lut) on the proliferation of U251 cells, Figure S4: The uncropped Western blots, Table S1: Genes altered in U251 Msi1 knockout cells, Table S2: Genes altered in U343 Msi1 knockout cells, Table S3: Genes altered in both U251 and U343 Msi1 knockout cells, Table S4: Gene Ontology and Pathway analyses of genes altered in both U251 and U343 Msi1 knockout lines, Table S5: Comparison between genes downregulated in both Msi1 knockout cells and genes upregulated in transgenic mouse lines expressing Msi1, Table S6: Genes showing expression high correlation with E2F2 and E2F8 in TCGA glioblastoma samples, Table S7: RNA-seq analysis of E2F2 and E2F8 knockdown in U251 cells, Table S8: Analysis of E2F8 target set, Table S9: List of primers used in qRT-PCR analysis.
