# Supplementary material for: Musashi1 Contribution to Glioblastoma Development via Regulation of a Network of DNA Replication, Cell Cycle and Division Genes
Source: Cancers (Basel). 2021 Mar 24;13(7):1494. doi: 10.3390/cancers13071494 (PMC8036803; doi:10.3390/cancers13071494)
Supplement: Supplementary file 1 [file cancers-13-01494-s001.zip › cancers-1120624-supplementary final/cancers-1120624-supplementary.pdf]

cancers
MDPI

Supplementary Material: Musashi1 Contribution to Glioblastoma Development via Regulation of a Network of DNA Replication, Cell Cycle and Division Genes

Mirella Baroni, Caihong Yi, Saket Choudhary, Xiufen Lei, Adam Kosti, Denise Grieshober, Mitzli Velasco, Mei Qiao, Suzanne S. Burns, Patricia R. Araujo, Talia DeLambre, Mi Young Son, Michelina Plateroti, Marco A. R. Ferreira, E. Paul Hasty and Luiz O. F. Penalva

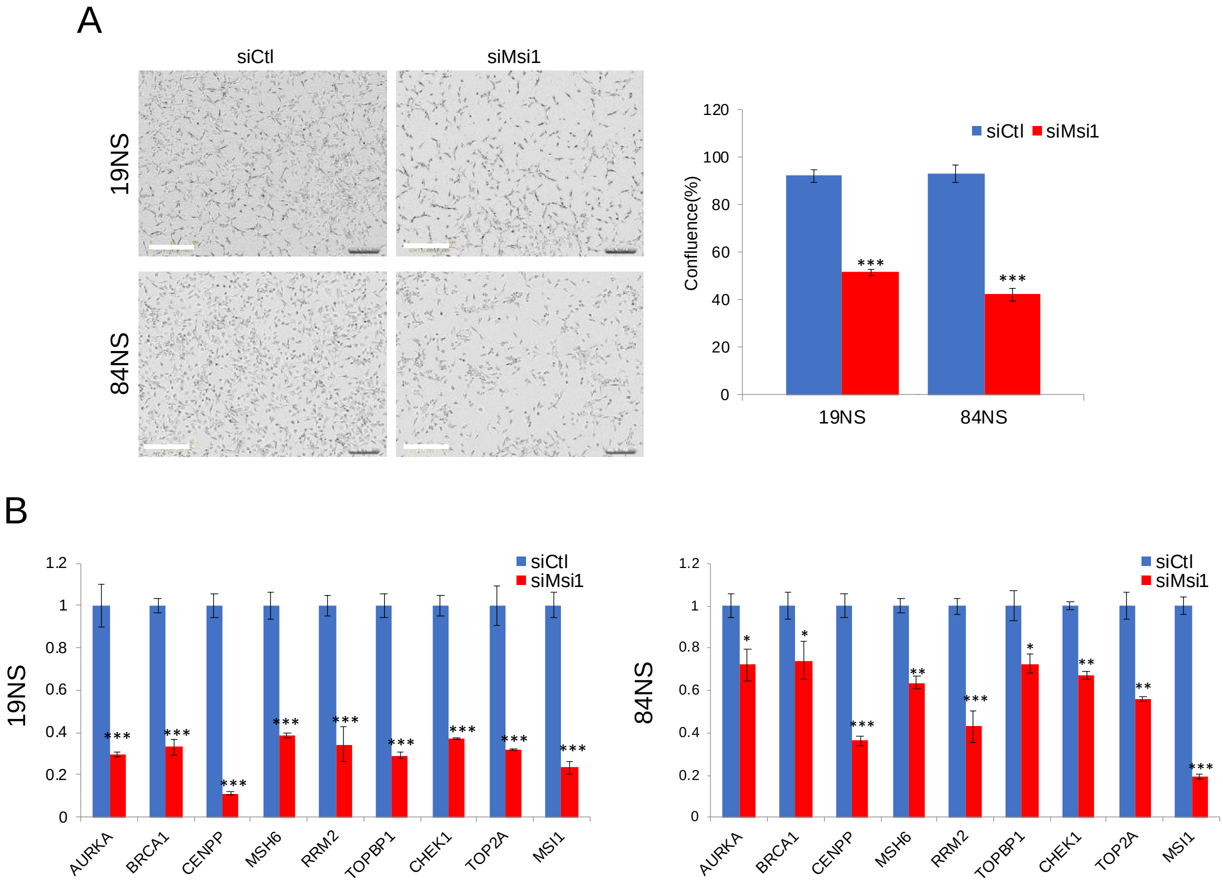

Figure S1. Msi1 knockdown suppressed GSC lines proliferation and affected the expression of cell cycle and DNA replication genes. (**A**) Images and bar graph of GSC lines showed reduced cell proliferation upon Msi1 knockdown. (**B**) qRT-PCR analysis of cell cycle and DNA replication genes in control and Msi1 knockdown GSCs. (* $p < 0.05$, ** $p < 0.01$, *** $p < 0.001$).

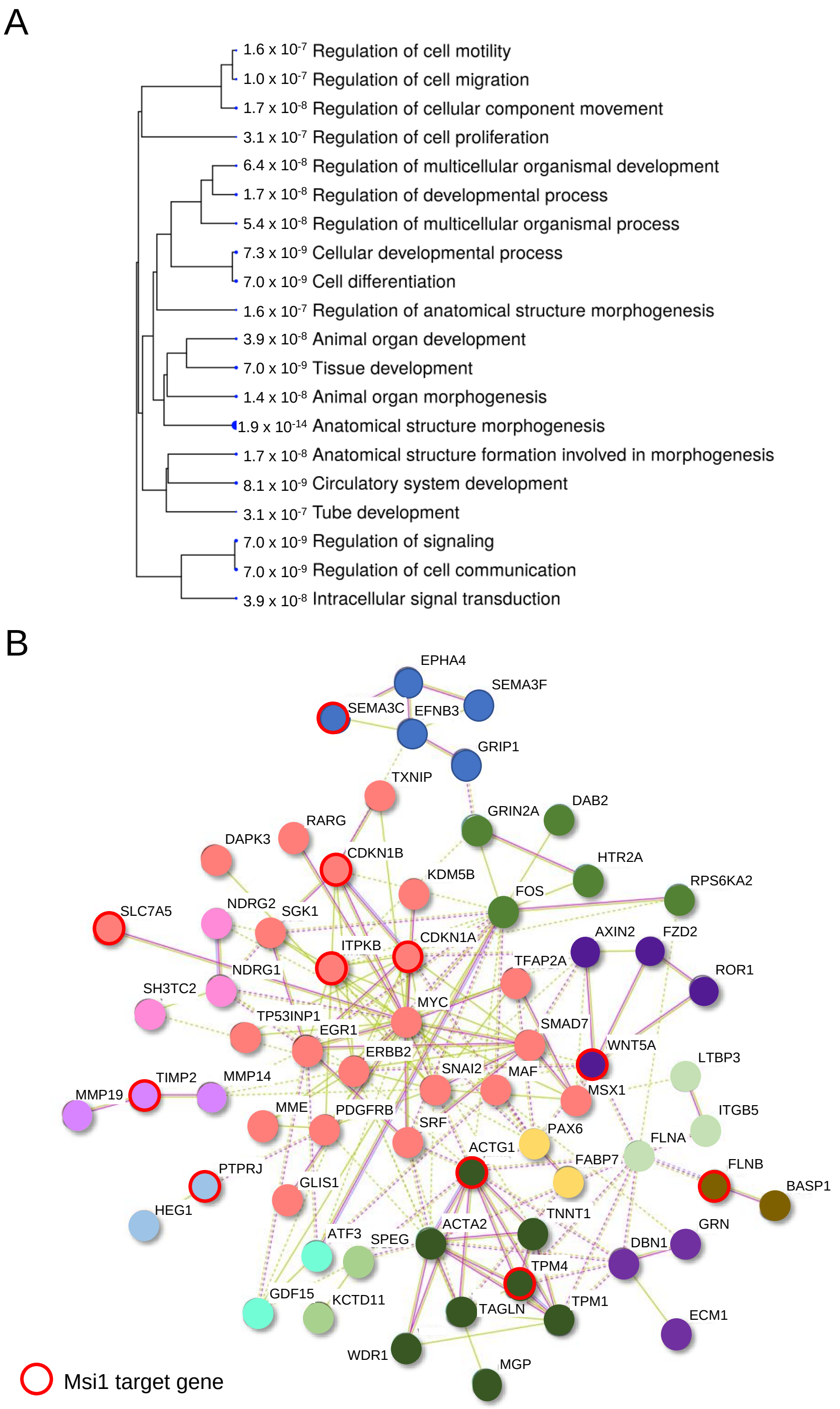

Figure S2. Glioblastoma cells with Musashi1 knockout show increased expression of a network of genes implicated in development and cell differentiation. (**A**) The diagram shows Gene Ontology terms (biological processes) enriched among genes upregulated after both U251 and U343 Msi1 knockout (KO) cells in comparison to respective controls, according to ShinyGo [1]. (**B**) Network shows genes implicated in development and differentiation that were upregulated in Msi1 KO cells of U251 and U343 lines in respect to control lines. The network was built using String [2] considering interaction (experimental evidence), text mining, and co-occurrence. Different colors were used to indicate clusters of highly associated genes.

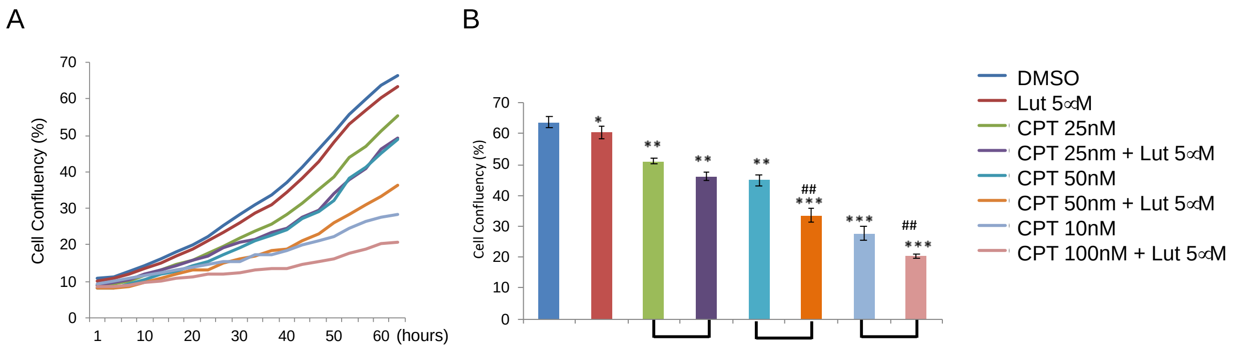

Figure S3. Synergistic effect of Camptothecin(CPT) and Luteolin (Lut) on the proliferation of U251 cells. U251 cells were plated onto 96-well plates (1500 cells/well). 24 hours later, cells were treated with a low concentration of luteolin or DMSO plus different concentrations of CPT. Plates were transferred to the IncuCyte and cells were counted every 4 hours for 4–6 days. (**A**) U251 cells proliferation with different combination of drug treatment over a period of 60 hours. (**B**) The graph shows side by side differences in proliferation between single and combined treatment at 60 hours. All experiments were performed in triplicate. The Combination Index (CI) was used to determine synergistic interaction. ## indicates cases when synergism was observed (##CI < 0.9). Statistical significance was calculated by t-test. All data are shown as means ± s.d. (*$p < 0.05$, ** $p < 0.01$, *** $p < 0.001$).

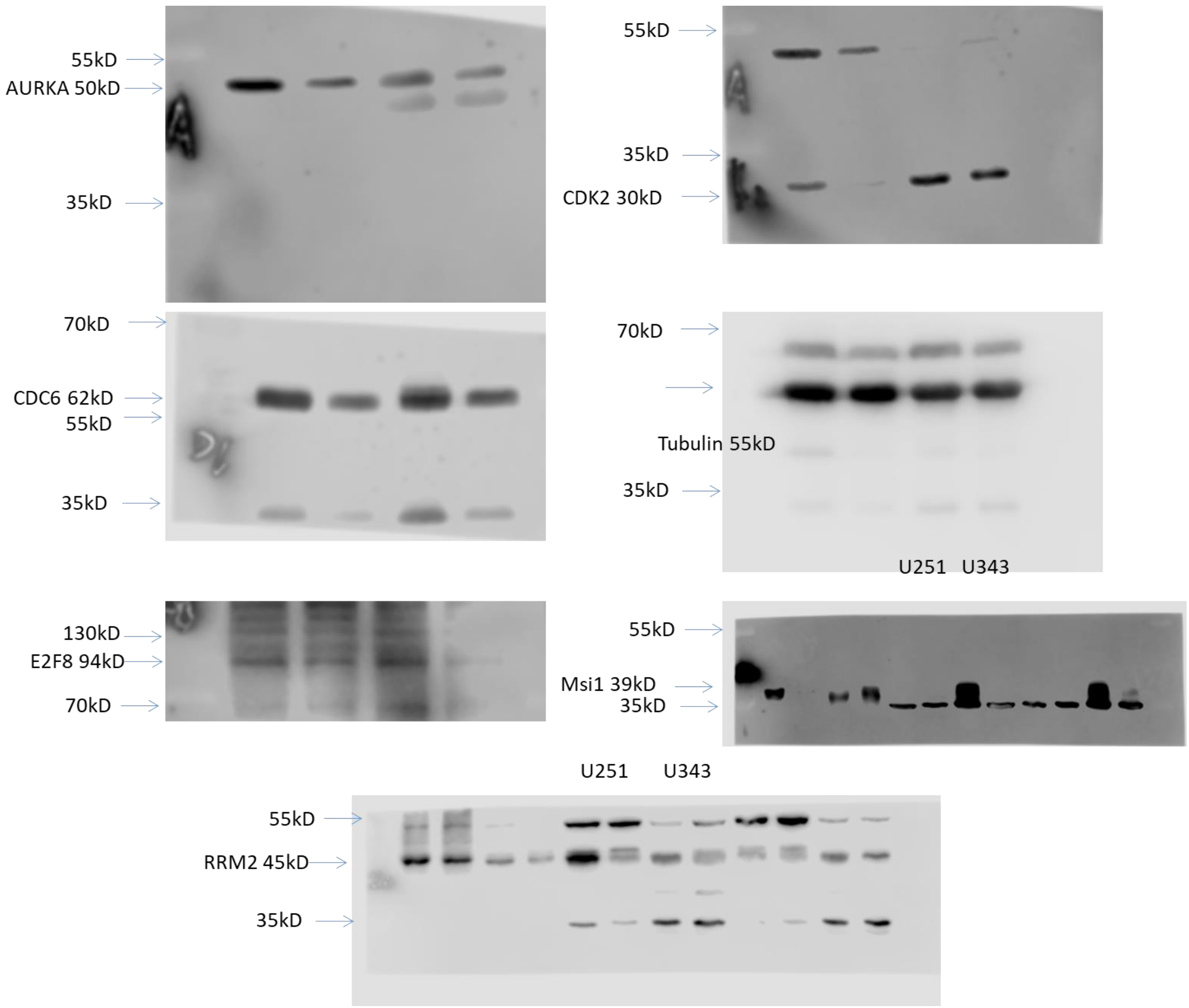

Figure S4. The uncropped Western blots.

Supplementary Tables

Table S1. Genes altered in U251 Msi1 knockout cells. **Sheet 1)** List of genes showing differential expression in two U251 Msi1 KO cells in comparison to control; **Sheet 2)** Gene Ontology analysis of downregulated genes in U251 Msi1 KO cells**; Sheet 3)** Pathway analysis of downregulated genes in Msi1 KO cells; **Sheet 4)** Gene Ontology analysis of upregulated genes in U251 Msi1 KO cells; **Sheet 5)** Pathway analysis of upregulated genes in U251 Msi1 KO cells.

Table S2. Genes altered in U343 Msi1 knockout cells. **Sheet 1)** List of genes showing differential expression in two U343 Msi1 KO cells in comparison to control; **Sheet 2)** Gene Ontology analysis of downregulated genes in U343 Msi1 KO cells**; Sheet 3)** Pathway analysis of downregulated genes in U343 Msi1 KO cells; **Sheet 4)** Gene Ontology analysis of upregulated genes in U343 Msi1 KO cells; **Sheet 5)** Pathway analysis of upregulated genes in U343 Msi1 KO cells.

Table S3. Genes altered in both U251 and U343 Msi1 knockout cells. **Sheet 1)** List of genes downregulated in both U251 Msi1 KO and U343 Msi1 KO cells in comparison to respective controls; **Sheet 2)** Detailed information for Sheet 1; **Sheet 3)** Comparison between genes downregulated in Msi1 KO cells and miR-137 targets [3]; **Sheet 4)** List of genes upregulated in both U251 Msi1 KO and U343 Msi1 KO cells in comparison to respective controls; **Sheet 5)** Detailed information for Sheet 4.

Table S4. Gene Ontology and Pathway analyses of genes altered in both U251 and U343 Msi1 knockout lines. **Sheet 1)** Gene Ontology analysis of downregulated genes in both Msi1 KO cells**; Sheet 2)** Pathway analysis of downregulated genes in both Msi1 KO cells; **Sheet 3)** Gene Ontology analysis of upregulated genes in both Msi1 KO cells; **Sheet 4)** Pathway analysis of upregulated genes in both Msi1 KO cells.

Table S5. Comparison between genes downregulated in both Msi1 knockout cells and genes upregulated in transgenic mouse lines expressing Msi1. **Sheet 1)** Genes upregulated in transgenic mouse lines expressing Msi1 [4] and Gene Ontology analysis. **Sheet 2)** Comparison between genes downregulated in both Msi1 KO cells and genes upregulated in transgenic mouse lines expressing Msi1. **Sheet 3)** Comparison between Gene Ontology analyses of genes downregulated in both Msi1 KO cells and genes upregulated in transgenic mouse lines expressing Msi1.

Table S6. Genes showing expression high correlation with E2F2 and E2F8 in TCGA glioblastoma samples. **Sheet 1)** Genes showing high expression correlation with E2F2 in TCGA GBM samples. **Sheet 2)** Genes showing high expression correlation with E2F8 in TCGA GBM samples. **Sheet 3)** Table shows genes downregulated in Msi1 KO cells that display expression correlation with E2F2 and E2F8.

Table S7. RNA-seq analysis of E2F2 and E2F8 knockdown in U251 cells. **Sheet 1)** Down-regulated genes in E2F2 knockdown cells in. **Sheet 2)** Down-regulated genes in E2F8 knockdown cells in comparison to control. **Sheet 3)** Overlap between genes downregulated in U251 E2F2 and E2F8 knockdown cells and Msi1 KO cells. **Sheet 4)** Consensus overlap and gene ontology analysis. **Sheet 5)** Up-regulated genes in E2F2 knockdown cells. **Sheet 6)** Up-regulated genes in E2F8 knockdown cells in comparison to control. **Sheet 7)** Overlap between genes up-regulated in E2F2 and E2F8 knockdown cells and Msi1 KO cells. **Sheet 8)** Consensus overlap and gene ontology analysis.

Table S8. Analysis of E2F8 target set. **Sheet 1)** Comparison between ENCODE E2F8 ChIP-seq data and genes downregulated in U251 upon E2F8 knockdown. **Sheet 2)** Summary of E2F8 ChIP-seq results and GO analysis. **Sheet 3)** GBM single cell analysis [5] showing E2F8 and genes downregulated in Msi1 KO cells in cluster linked to cell cycle (cluster 1).

Table S9. List of primers used in qRT-PCR analysis.

References

1. Ge, S.X.; Jung, D.; Yao, R. ShinyGO: a graphical gene-set enrichment tool for animals and plants. *Bioinformatics* **2020**, *36*, 2628–2629, doi:10.1093/bioinformatics/btz931.

2. Szklarczyk, D.; Gable, A.L.; Lyon, D.; Junge, A.; Wyder, S.; Huerta-Cepas, J.; Simonovic, M.; Doncheva, N.T.; Morris, J.H.; Bork, P., et al. STRING v11: protein-protein association networks with increased coverage, supporting functional discovery in genome-wide experimental datasets. *Nucleic. Acids. Res.* **2019**, *47*, D607–D613, doi:10.1093/nar/gky1131.
3. Velasco, M.X.; Kosti, A.; Guardia, G.D.A.; Santos, M.C.; Tegge, A.; Qiao, M.; Correa, B.R.S.; Hernández, G.; Kokovay, E.; Galante, P.A.F., et al. Antagonism between the RNA-binding protein Musashi1 and miR-137 and its potential impact on neurogenesis and glioblastoma development. *RNA* **2019**, *25*, 768–782, doi:10.1261/rna.069211.118.
4. Cambuli, F.M.; Correa, B.R.; Rezza, A.; Burns, S.C.; Qiao, M.; Uren, P.J.; Kress, E.; Boussouar, A.; Galante, P.A.; Penalva, L.O., et al. A Mouse Model of Targeted Musashi1 Expression in Whole Intestinal Epithelium Suggests Regulatory Roles in Cell Cycle and Stemness. *Stem. Cells* **2015**, *33*, 3621–3634, doi:10.1002/stem.2202
5. Pang, B.; Xu, J.; Hu, J.; Guo, F.; Wan, L.; Cheng, M.; Pang, L. Single-cell RNA-seq reveals the invasive trajectory and molecular cascades underlying glioblastoma progression. *Mol. Oncol.* **2019**, *13*, 2588–2603, doi:10.1002/1878-0261.12569.
